# A modified and tailored human follicle isolation procedure improves follicle recovery and survival

**DOI:** 10.1186/s13048-017-0366-8

**Published:** 2017-10-23

**Authors:** Maria Costanza Chiti, Marie-Madeleine Dolmans, Maria Hobeika, Alice Cernogoraz, Jacques Donnez, Christiani Andrade Amorim

**Affiliations:** 1 Pôle de Recherche en Gynécologie, Institut de Recherche Expérimentale et Clinique, Université Catholique de Louvain, Avenue Mounier 52, bte. B1.52.02, 1200 Brussels, Belgium; 2 Gynecology Department, Cliniques Universitaires Saint-Luc, 1200 Brussels, Belgium; 3 Society for Research into Infertility, Brussels, Belgium

**Keywords:** Ovarian follicle isolation, Xenografting, Fibrin matrix, Artificial ovary

## Abstract

**Background:**

Ovarian tissue cryopreservation followed by transplantation after cancer remission is the most commonly applied fertility restoration approach in very young girls and women who require immediate cancer therapy. However, clinicians strongly advise against reimplantation of one’s own ovarian tissue when there is a high risk of recurrence after grafting. For these patients, development of an alternative strategy, namely a transplantable artificial ovary, offers future hope of conceiving. The first essential requirement for an artificial ovary is the set-up of a safe and effective follicle isolation procedure. Despite encouraging results with different variants of this technique, none of them take into the account the physiology and great variability in follicular density inside individual tissue fragments and between different patients. The goal of this study was to improve our previously applied follicle isolation procedure in order to develop a tailored isolation procedure for human follicles according to individual tissue properties. To this end, enzymatic digestion was divided into three time intervals in order to initially recover the first follicles to be isolated, and then further dissociate undigested fragments of tissue containing entrapped follicles.

**Results:**

After thawing frozen human ovarian tissue using a modified and tailored follicle isolation method, already 35% of follicles were fully isolated and recovered after 30 min of enzymatic digestion. Indeed, this protocol resulted in a higher follicle yield (*p* < 0.01) and greater numbers of primordial and primary follicles (*p* < 0.05) than the previous approach. However, no significant difference was found in caspase-3-positive and Ki67-positive staining between the two isolation protocols. In addition, greater follicle quality was demonstrated. When human follicles isolated using the modified protocol were encapsulated in a fibrin matrix with high concentrations of fibrinogen and thrombin and xenografted to a SCID mouse, more follicles were found to be healthy after one week of transplantation than in a previous our study.

**Conclusions:**

With the modified follicle isolation method, we were able to maximize the number and quality of isolated primordial and primary follicles, and develop a tailored follicle isolation procedure according to individual tissue properties. Moreover, improved follicle survival inside an artificial ovary prototype was detected after one week of xenografting.

**Electronic supplementary material:**

The online version of this article (10.1186/s13048-017-0366-8) contains supplementary material, which is available to authorized users.

## Background

In recent years, we have been focusing our efforts on the development of a transplantable artificial ovary, with a view to restoring fertility in cancer patients at high risk of ovarian involvement who cannot undergo any of the currently implemented fertility preservation strategies [1–3]. Conceptually, the artificial ovary would require removal and cryopreservation of ovarian tissue before initiation of cancer treatment, as with ovarian tissue transplantation [4–6], but unlike the latter, fragments of cryopreserved ovarian tissue would be thawed and mechanically and enzymatically digested in order to isolate preantral follicles once the patient is cured. Thereafter, these follicles would be encapsulated in a three-dimensional (3D) scaffold and transplanted back to the patient in order to restore her fertility [7].

It is therefore clear that the first crucial step in such a procedure is effective isolation of human preantral follicles, which should constitute a perfect balance in terms of quantity and quality. An optimal isolation procedure should allow recovery of a maximum number of follicles from the ovarian tissue, without causing them any damage. Previous reports have already demonstrated that structurally intact human follicles can be isolated [8–12] and shown to be free of malignant cell contamination, an essential requirement for future clinical application of an artificial ovary [13, 14]. However, such protocols do not take into account the great heterogeneity of follicular density [15, 16] and extracellular matrix composition [17, 18] between patients, which inevitably influences isolation procedure success rates. Furthermore, enzymatic digestion is usually performed over a single duration of time, without considering the possible negative impact of prolonged digestion on the first fully isolated follicles. With this in mind, the aim of the present study was to improve the follicle isolation procedure in order to maximize the number and quality of isolated follicles, and develop a tailored follicle isolation approach according to each individual ovarian tissue sample.

## Methods

### Experimental design

Preantral follicles were isolated from human frozen-thawed biopsies (*n* = 23) according to two different isolation protocols (previously established versus modified) in order to compare follicle yield and follicle viability. Thereafter, between 14 and 50 isolated follicles (from each protocol) were embedded in fibrin clots composed of 30–75 mg of fibrinogen (F) and 50–75 IU/mL of thrombin (T) to compare follicle developmental stage and survival. In addition, to confirm the impact of the modified isolation protocol on the survival of healthy follicles, one fibrin clot containing 100 follicles and 100,000 ovarian stromal cells (SCs) was xenografted to the ovarian bursa of a severe combined immunodeficient (SCID) mouse for 7 days.

### Thawing of frozen ovarian cortex and sample distribution

Cryovials of frozen ovarian cortex from the different patients (*n* = 23) were collected from the ovarian tissue bank and thawed as previously described [19]. Thawed ovarian fragments were measured using graph paper and then equally distributed based on their size between the previously established protocol and the newly modified version.

### Follicle isolation

#### Previously applied protocol

This protocol has already been extensively described [10] and is detailed in the Additional file 1. Briefly, it involves a single duration (75 min) of mechanical and enzymatic tissue digestion with 0.28 Wünsch units/mL Liberase DH (Roche Diagnostics, GmbH, Mannheim, Germany) before follicle pick-up.

#### Modified protocol

In the modified version, ovarian tissue pieces were mechanically minced into fragments with a tissue chopper (McIlwain Tissue Chopper, Mickle Laboratory, Guildford, UK) and incubated in 10 mL of Dulbecco’s phosphate-buffered saline (PBS) with Ca^2+^ and Mg^2+^ (Gibco, Thermo Fisher Scientific, Ghent, Belgium) in the presence of 0.28 Wünsch units/mL Liberase DH (Roche Diagnostics) and 8 Kunitz units/mL DNase I (Roche Diagnostics, Brussels, Belgium) in a water bath (37 °C) for 30 min with gentle agitation and pipetted every 15 min. After 30 min, the suspension was first filtered with a cell strainer of 70 μm (Falcon, New York, USA), then 30 μm (pluriSelect Life Science, Leipzig, Germany), before being inactivated with the same volume of PBS + 10% heat inactivated fetal bovine serum (HIFBS). The enzymatic digestion step was repeated with ovarian tissue fragments collected from the 70 μm cell strainer, while isolated follicles from the 30 μm filter were recovered by washing the nylon mesh in 3 mL of PBS + 10% HIFBS. Fully isolated follicles were retrieved with the help of a stereomicroscope and 130 μm micropipette by two operators. Every 30 min, the filtration and follicle recovery steps were repeated. Once 90 min of enzymatic digestion was reached, with virtually all ovarian tissue fragments totally dissociated, the isolation procedure was halted. It is important to mention that each filter was checked under the stereomicroscope for entrapped follicles possibly remaining in the nylon mesh, before being discarded.

### Ovarian SC isolation

After follicle isolation, the remaining ovarian stromal cell suspension was filtered through sterilized 11-μm nylon net filters (Millipore, Overijse, Belgium). A volume of 10 μL of filtered solution was analyzed using Trypan blue (Sigma-Aldrich), and a Bürker chamber (VWR, Leuven, Belgium) was utilized to count cell numbers. Thereafter, the filtered solution was centrifuged at 260 *g* for 5 min and the pellet resuspended in PBS supplemented with 10% FBS to obtain a concentration of 100,000 cells/1 μL.

### Follicle yield

Because of the diameter of the polycarbonate needle (130 μm) used to pick up follicles in both the established and optimized protocols, follicle diameter ranged between 30 and 129 μm. The chance of finding follicles larger than 120 μm is actually very low when we perform human ovarian follicle isolation from frozen-thawed fragments of ovarian tissue. Isolated preantral follicles were retrieved and counted in both protocols in order to compare their number and quality.

### Follicle viability

To assess follicle viability, 5 to 10 follicles were incubated in 50 μL of PBS containing 2 μmol/L calcein AM and 5 μmol/L ethidium homodimer-I (LIVE/DEAD® Viability/Cytotoxicity Kit, Molecular Probes, Leyden, the Netherlands) for 30 min at 37 °C in the dark [9]. The follicles were then classified into four categories (V1 to V4) depending on the percentage of dead granulosa cells (GCs) [20]: V1) follicles with all GCs viable; V2) follicles with less than 10% dead GCs; V3) follicles with 10–50% dead GCs; and V4) follicles with more than 50% dead GCs and/or a dead oocyte. V1 and V2 were considered to be live follicles, V3 and V4 were classified as dead.

### Fibrin clot reconstitution and follicle encapsulation

Reconstitution and dilution of the two components (F and T) of the fibrin sealant kit (Tissucol, Baxter, Lessines, Belgium) and fibrin clot formation were previously described by Paulini et al. [21]. F (100 mg/mL) was diluted in saline solution (0.9% NaCl) to obtain a final concentration ranging from 30 to 75 mg/mL, and T (500 IU/mL) was diluted in 40 mmol/L CaCl_2_ to achieve a concentration between 50 and 75 IU/mL.

### Immunohistochemistry

To evaluate follicle health status after isolation and fibrin encapsulation, we performed caspase-3 and Ki67 immunostaining [22, 23]. The protocol is described in detail in the Additional file 1. Follicles with more than 50% of GCs and/or the oocyte positive for caspase-3 were classified as atretic, while follicles with at least one Ki67-marked GC were considered to be growing [21].

### Xenografting of a fibrin clot to a SCID mouse

In order to confirm the effectiveness of the modified protocol, a fibrin clot containing 100 follicles and 100,000 SCs was xenografted to the left ovarian bursa of a SCID mouse. Guidelines for animal welfare were approved by the Committee on Animal Research of the Université Catholique de Louvain. The mouse was anesthetized according to a previous protocol [24, 25]. A medio lateral dorsal cutaneous incision was made and the peritoneum was opened through a small hole. The ovary was exposed and the periovarian fat was gently gripped with forceps in order to stabilize the ovary and give access to the ovarian bursa. Under an operating microscope (Leica MZ6, Wetzlar, Germany), a small opening was made in the ovarian bursal membrane to introduce the fibrin clot, before closing the membrane with non-absorbable 8/0 Prolene suture (Ethicon, Johnson & Johnson Medical, Livingston, UK). The adnexa were replaced inside the peritoneal cavity and the peritoneum and skin were closed with non-absorbable 6/0 Prolene. After surgery, anesthesia was reversed as previously described [24, 25]. The animal was euthanized after 7 days by cervical dislocation and the graft was recovered and fixed in 4% formaldehyde.

### Histological analysis

After 24 h of fixation in formaldehyde, the fibrin clots were embedded in paraffin for histological analysis. Each fibrin clot was cut into 5 μm serial sections and every second section was stained with hematoxylin and eosin to be counted and classified according to the developmental stage of the encapsulated follicles [24].

Follicles were classified as primordial when a single layer of flattened GCs was observed surrounding the oocyte, intermediate when one layer of flattened and cuboidal GCs was detected, primary when a full layer of cuboidal GCs was identified around the oocyte, and secondary when two or more layers of cuboidal GCs were found [26].

### Statistical analysis

The Wilcoxon test was used to compare follicle yield and viable follicles before encapsulation between the previous and the modified isolation protocol. Fisher’s exact test was applied to compare follicle developmental stages and Ki67-positive follicles after encapsulation. A *p*-value <0.05 was considered statistically significant. All statistical analyses were performed with GraphPad Prism 7 software (GraphPad Software, La Jolla, USA).

## Results

### After isolation

#### Follicle yield

**Fig. 1:**
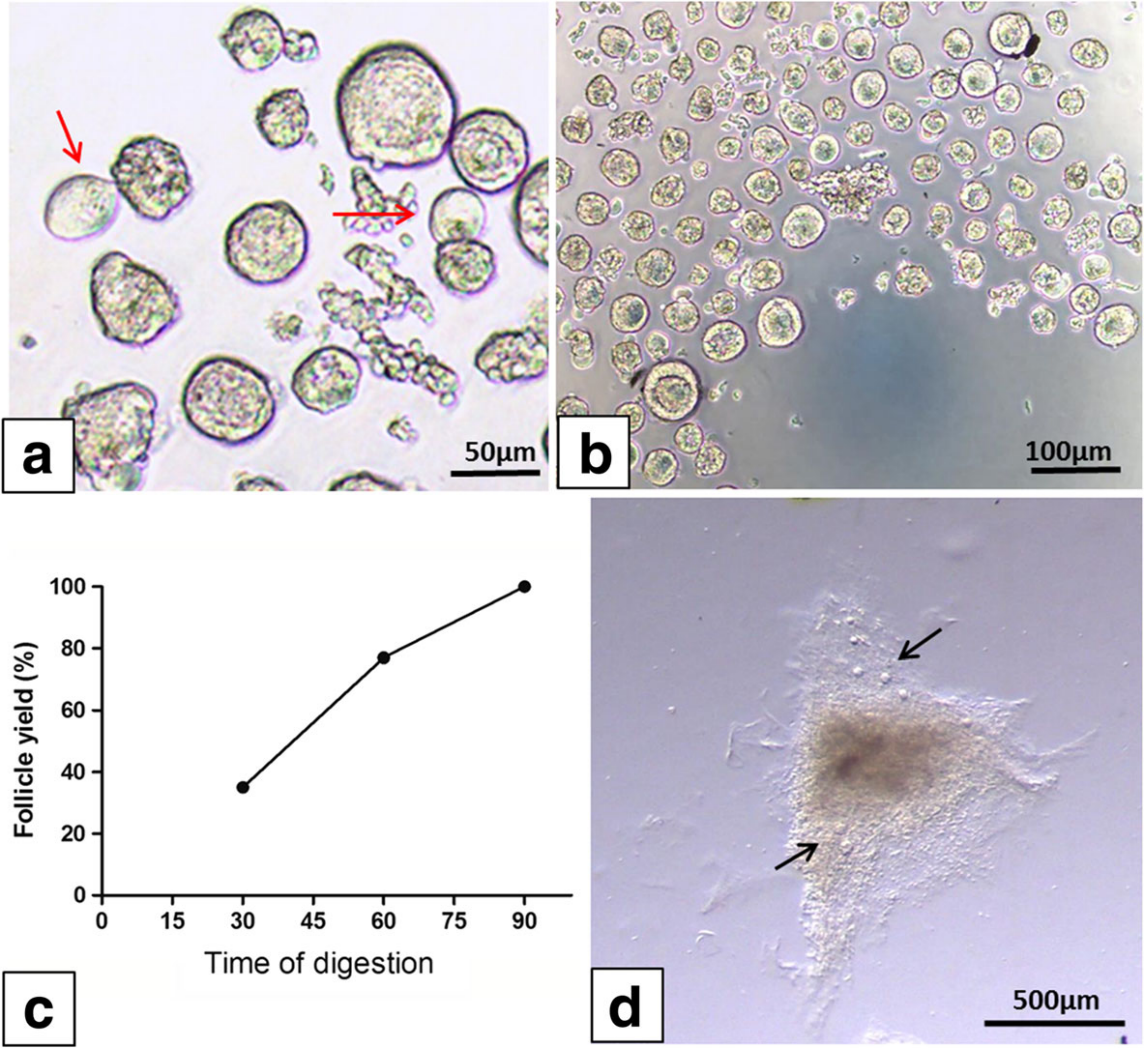
Isolation of human preantral follicles. In the previous protocol, some follicles showed extruded oocytes (red arrow) (**a**); in the modified protocol, the vast majority of follicles were isolated from ovarian tissue samples (**b**). Distribution of isolated follicles in the modified protocol according to the three time intervals (**c**); some follicles remained entrapped in fragments of undigested tissue (black arrows) (**d**)

A total of 4661 follicles were isolated with the two protocols, 1938 with the previously applied protocol after 75 min of digestion (Table 1, Fig. 1a), and 2723 with the modified protocol using the same sample size from the same patients (Table 1, Fig. 1b).

**Table 1:** Follicle yield according to follicle isolation protocol. Individual follicle yield from 22 patients according to different follicle isolation protocols

Patient	Established	Modified
1	2	11
2	23	93
3	45	61
4	162	117
5	152	272
6	50	105
7	110	90
8	0	0
9	341	483
10	200	303
11	7	18
12	13	19
13	105	177
14	125	135
15	58	70
16	7	28
17	29	65
18	17	15
19	105	91
20	65	119
21	125	127
22	197	324
Total	1938	2732

Moreover, in the latter case, 35% of that number were already fully isolated and recovered after 30 min of digestion. The remaining 65% were recovered after 60 min (42%) and 90 min (23%) of digestion (Fig. 1c). As already well known from the literature [16], there is great variation in follicle density between patients (Table 1). When the mean ± SD of isolated follicles was compared between the two protocols, the modified version showed a significantly higher number of isolated follicles (123.8 ± 121.7) than the previous protocol (88.1 ± 85.4) (*p* < 0.01). In some cases, residual fragments of undigested tissue containing follicles were found with both protocols (Fig. 1d).

#### Follicle viability

Shortly after isolation, a total of 237 follicles were analyzed for viability (115 and 122 follicles with the previously applied and modified protocols respectively). The percentage of viable and minimally damaged follicles (V1 and V2) was high and similar between the two isolation protocols (85% and 96% for old and new respectively). Hence, the percentage of damaged and dead follicles (V3 and V4) was low and also comparable (15% and 4% respectively). The number of live follicles (V1 and V2) was therefore significantly greater than the number of dead follicles with both isolation protocols (*p* < 0.0001).

### After encapsulation

#### Follicle counting and classification

**Fig. 2:**
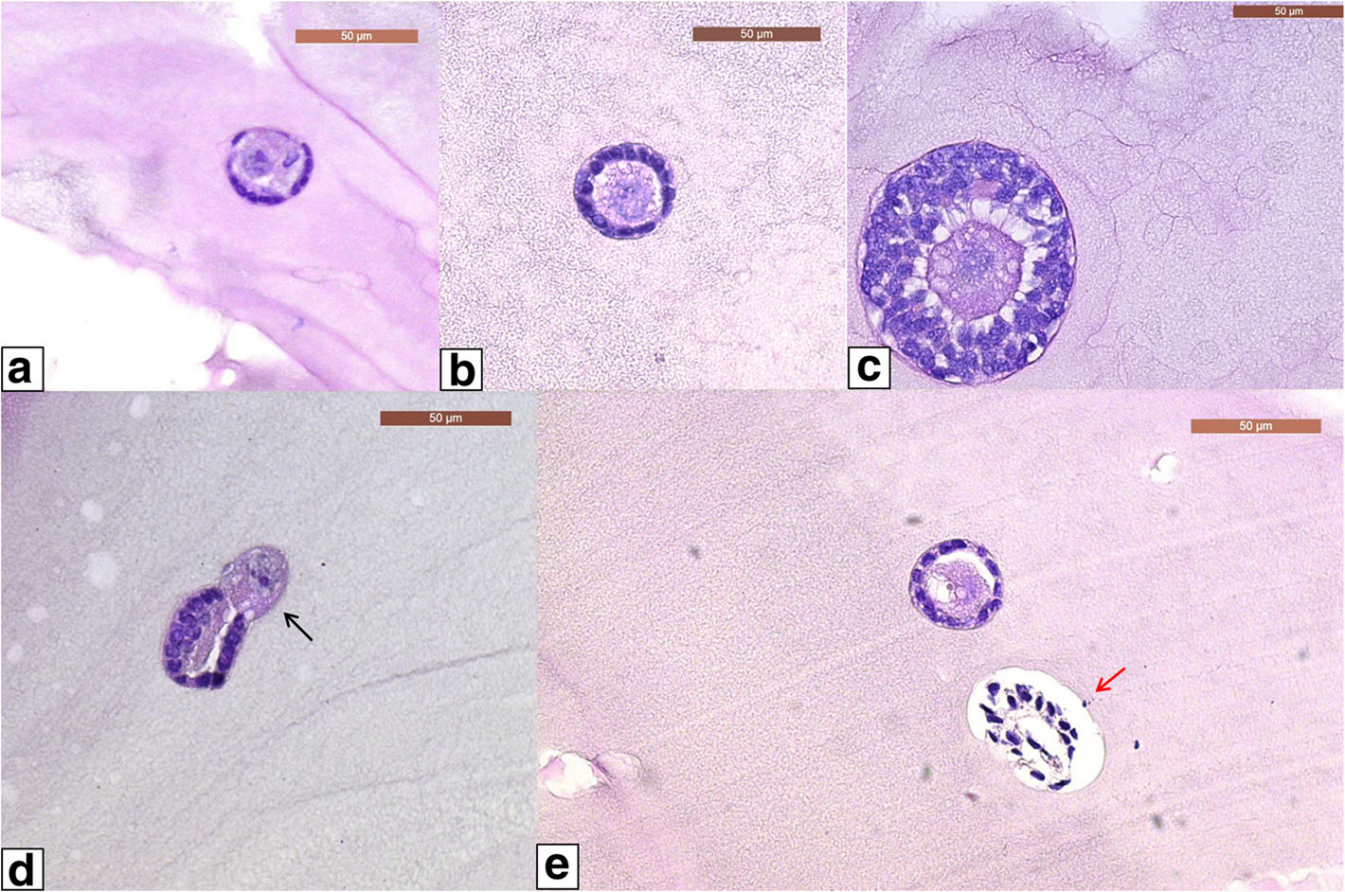
Histologically normal representations of human follicles encapsulated in a fibrin matrix. Primordial (**a**), primary (**b**) and secondary (**c**) follicles after isolation with the modified protocol and encapsulation in a fibrin matrix. An extruded oocyte (black arrow) (**d**) and normal and degenerated follicles (red arrow) were found with the previous protocol after histological analysis (**e**)

A total of 22 fibrin clots (*n* = 10 with the previous protocol and *n* = 12 with the modified protocol) were processed for hematoxylin and eosin staining and compared according to follicle developmental stage (primordial, primary or secondary). As shown in Table 2, the modified protocol yielded significantly higher number of primordial and primary follicles (*p* < 0.05) and a significantly lower number of secondary follicles (*p* < 0.01) than the previous protocol. Primordial, primary and secondary follicles are illustrated after isolation and fibrin encapsulation in Fig. 2.

**Table 2:** Follicle developmental stage after fibrin matrix encapsulation. Follicle developmental stages were compared between the previous and modified isolation protocols

Number of follicle stage after fibrin encapsulation		
	Previous protocol (%)	Modified protocol (%)
Primordial	14 (7%) ^a^	18 (15%) ^b^
Primary	127 (66%) ^a^	90 (77%) ^b^
Secondary	53 (27%) ^c^	9 (8%) ^d^

Different letters (a, b, c, d) in each row indicate significant differences (a, b *p* < 0.05; c, d *p* < 0.01)

#### Follicle health status

**Fig. 3:**
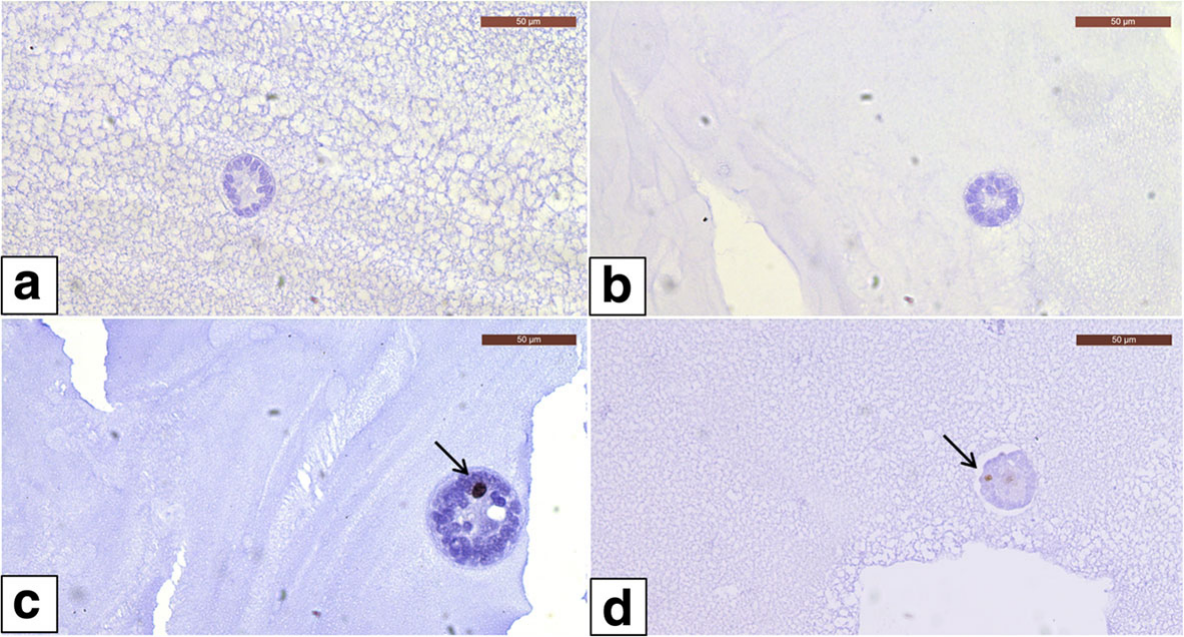
Follicle health status after caspase-3 and Ki67 immunohistochemistry. Caspase-3-negative primary follicles isolated with the established (**a**) and modified (**b**) isolation protocols. Ki67-positive follicles isolated with the established (**c**) and modified (**d**) isolation protocols

Of 25 follicles (previous protocol *n* = 9; modified protocol *n* = 16) analyzed for caspase-3, none were positive, which proves that 100% of evaluated follicles were alive soon after isolation and encapsulation, irrespective of isolation protocol (Fig. 3a, b).

A total of 44 follicles were analyzed for Ki67 (previous protocol *n* = 29; modified protocol *n* = 15). With the previous protocol, 8 follicles (27.6%) were Ki67 positive, 3.4% of which were primordial (n = 1), 10.3% primary (*n* = 3), and 13.8% secondary follicles (*n* = 4) (Fig. 3c). With the modified protocol, only 1 follicle (6.7%) was found to be positive for Ki67 and it was at the primary-secondary stage (Fig. 3d). However, no difference was found in the number of Ki67 positive follicles between the two protocols (Table 3).

**Table 3:** Distribution of Ki67-positive and -negative follicles according to the established and modified isolation protocols. Follicle developmental stages were compared between the previous and modified isolation protocols

Follicle stage	Established (*n* = 29)		Modified (*n* = 15)		*p*-value
	Ki67 +	Ki67 -	Ki67 +	Ki67 -	
Primordial	1^a^	28	0^a^	15	1.000
	(3.4%)		0%		
Primary	3^b^	26	0^b^	15	0.5402
	(10.3%)		0%		
Secondary	4^c^	25	1^c^	14	0.6467
	(13.8%)		6.7%		

Different letters (a, b, c) in each row indicate significant differences (a, b, c *p* < 0.05)

### After grafting

#### Follicle recovery rate

**Fig. 4:**
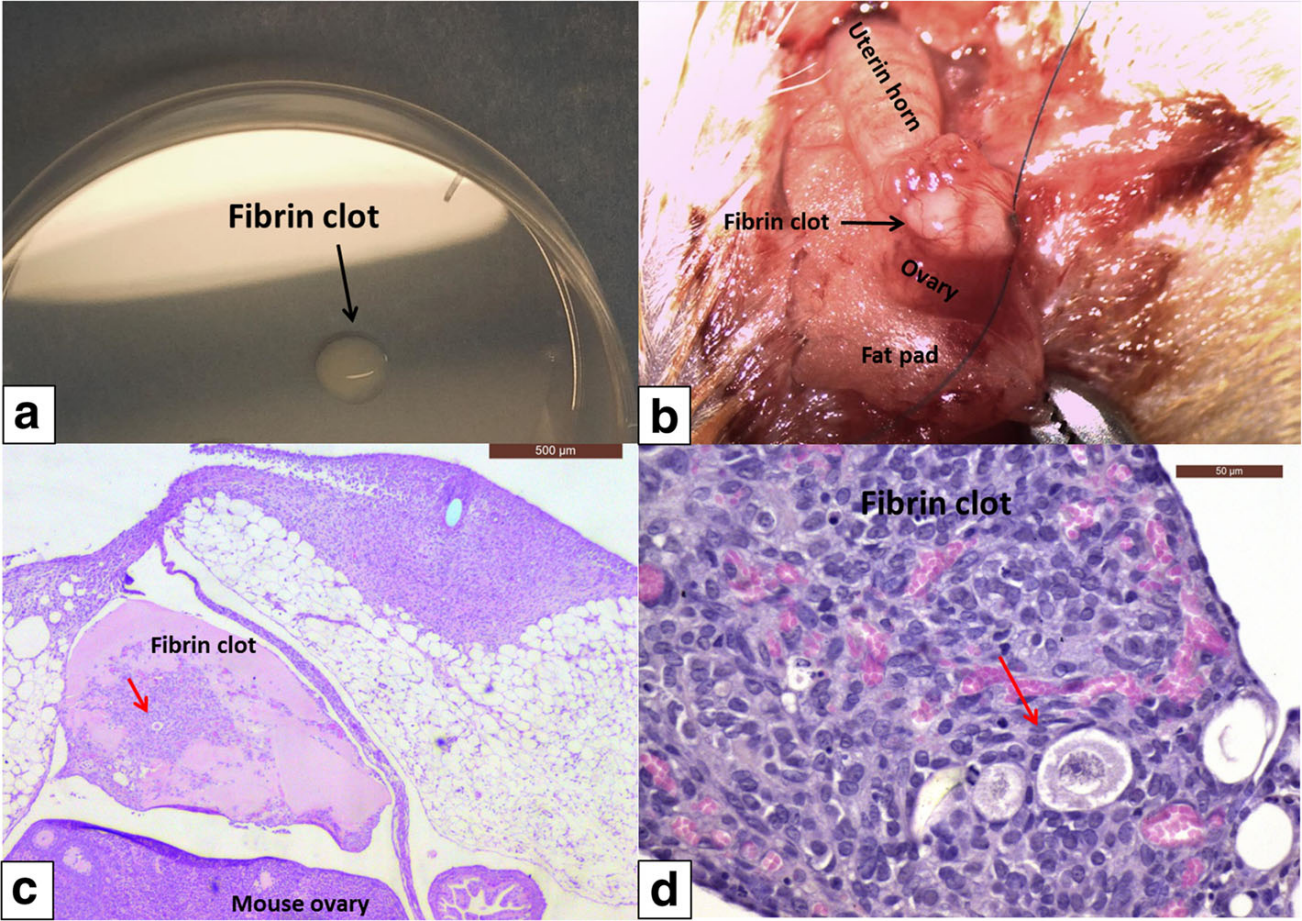
Grafting of a fibrin clot to the ovarian bursa of a SCID mouse. A fibrin clot containing human follicles and ovarian SCs isolated using the modified protocol before transplantation (**a**); xenografting of the fibrin clot to the ovarian bursa of a SCID mouse (**b**); histological representation of the fibrin clot inside the ovarian bursa of a SCID mouse after 7 days of transplantation: a follicle is visible in the middle of the fibrin clot (red arrow) (**c**); histological representation of follicles found in fibrin clot after 7 days of transplantation (red arrow) (**d**)

Since around 50% of encapsulated follicles are found soon after fibrin polymerization and histological investigation [21, 25], in order to evaluate the real number of follicles remaining after transplantation, we applied a correction factor as previously reported [21, 25]. Consequently, 18 out of 50 follicles (35%) were identified on day 7 of transplantation. Of this number, 72% and 28% were at the primordial and primary stage respectively. Figure 4 shows some of these recovered follicles after 7 days of grafting.

## Discussion

Aiming to create a transplantable artificial ovary, our goal was to optimize our previous follicle isolation protocol in order to increase the number and survival of isolated human preantral follicles [9, 10]. While the previous method does yield a high number of isolated preantral follicles, we always observed extruded oocytes [9, 10] and undigested fragments of tissue. This was probably due to the prolonged and fixed duration of enzymatic digestion (75 min). For this reason, we hypothesized that a successful isolation protocol should take into account two important points: first, the high degree of heterogeneity in follicle density, not only between women and age groups [13, 25], but even between fragments of ovarian tissue from the same patient [14]; and second, the variations in extracellular matrix composition between women of different age [15, 16] and health status [15, 18] and even between different areas of the same ovarian tissue sample [16, 27], which influences success rates of the isolation procedure. To meet these requirements, the follicle isolation protocol should be tailored to each patient; instead of a fixed period for enzymatic digestion, as currently reported in the literature [7–11], this step would be halted as soon as all the fragments are totally digested. In addition, enzymatic digestion would be divided into several time intervals, in order to recover already isolated follicles, which would reduce the proportion of damaged follicles due to prolonged digestion. Therefore, with a view to maximizing the yield and quality of isolated ovarian follicles for each patient, we developed a tailored follicle isolation protocol that addresses the two limitations encountered with our earlier method [9, 10], namely long and fixed enzyme exposure time. Comparison of the two isolation protocols showed that with our modifications, based on fractioning enzymatic digestion over three different durations, we could obtain a greater number of isolated follicles than with the previously applied protocol. Since 35% of isolated follicles were already identified after the initial 30 min of enzymatic digestion in the modified protocol, we can confirm our hypothesis that the long period of enzyme exposure with the previous method has a negative impact on follicles. Although dispase present in high concentrations in Liberase DH does not cleave laminin [28], which is one of the main components of the basement membrane of human primordial follicles [29], it cleaves collagen IV [28], another essential constituent of the basement membrane of these follicles [29]. Hence, one can assume that follicles isolated during the first few minutes of enzymatic digestion could have their basement membrane progressively damaged over the course of 75 min of exposure to the enzyme, which would probably explain the presence of extruded oocytes in our previous studies [9, 10]. As also demonstrated by histological analysis, the number of primordial and primary follicles was significantly higher after fibrin encapsulation in the modified protocol (92.3%) than in the earlier one (73.4%), which more closely reflects the physiological distribution of follicles inside ovarian cortex [19, 30]. Our findings therefore confirm that discontinuous enzymatic digestion positively affects recovery rates of isolated follicles by protecting their structure from unnecessary exposure to Liberase DH. Interestingly, the percentage of secondary follicles was greater with the previous method than the modified version (26% versus 9%), but similar to an earlier report [12]. Unlike in the previous protocol, a filtration step was also added to the new protocol in order to replace follicle centrifugation and separate follicles from ovarian cell suspensions. We could hypothesize that the different percentage of secondary follicles found between the two protocols may be ascribed to some secondary follicles remaining trapped inside the filter and thus getting lost. With the modified isolation protocol, we even recovered a significant number of viable isolated follicles after 90 min of enzymatic incubation, which shows that the ovarian tissue fragments that remained undigested in our previous protocol are actually an important but as yet unexploited source of primordial follicles. Since every follicle counts towards increasing the likelihood of pregnancy in patients and the possibility of repeating this strategy, discarding such precious material may have a negative impact on artificial ovary outcomes. Another important point to bear in mind is that the artificial ovary will be mostly applied to prepubertal cancer patients. The ovaries of these young girls have a softer cortex than that of adult women, since the ovaries accumulate collagen fibers with age [31, 32] making the cortex stiffer. It would therefore be logical to assume that follicle isolation should be considerably faster in young patients than in adult women [13], so it is likely that the 75-min digestion time in our previous protocol would damage a significant number of these follicles. In order to confirm the effectiveness of the modified isolation protocol for our artificial ovary prototype, one fibrin clot containing isolated follicles and SCs was grafted for a short period of time. Interestingly, with the modified protocol, the superior follicle recovery rate after grafting (35%) was better than in a previous study (20–23%), where follicles were isolated using the earlier protocol and grafted inside a fibrin-based matrix [21] for one week. Moreover, in the present study, a higher percentage of primordial follicles (72% versus 15%) but a similar proportion of primary follicles (28% versus 26%) were found on day 7 of transplantation compared to our previous findings [21]. Given the superior preservation of the primordial follicle pool with the modified protocol, it could be interesting to further investigate whether this protocol plays a role in post-grafting activation of follicles.

## Conclusions

In conclusion, this study describes a personalized isolation procedure for human preantral follicles, which will allow us to maximize the number and quality of isolated primordial and primary follicles. Indeed, fractionating enzymatic digestion yielded a greater number of recovered follicles. Moreover, this protocol can be adapted to each patient, taking into account the density of their ovarian tissue. Therefore, with a view to clinical application of artificial ovary transplantation, we suggest systematic implementation of a tailored follicle isolation approach.

## Additional file

Additional file 1: Supplementary material. (DOCX 18 kb)

## Abbreviations

3D: Three-dimensional
F: Fibrinogen
GC: Granulosa cell
HIFBS: Heat-inactivated fetal bovine serum
PBS: Phosphate-buffered saline
RT: Room temperature
SC: Ovarian stromal cell
T: Thrombin
