# Supplementary material for: A modified and tailored human follicle isolation procedure improves follicle recovery and survival
Source: J Ovarian Res. 2017 Oct 23;10:71. doi: 10.1186/s13048-017-0366-8 (PMC5654051; doi:10.1186/s13048-017-0366-8)
Supplement: Supplementary file 1 — Supplementary material. (DOCX 18 kb) [file 13048_2017_366_MOESM1_ESM.docx]

**Additional file**

Follicle isolation

*Previous protocol*

Thawed ovarian cortex biopsies were first mechanically and then enzymatically digested, before being minced into fragments with a tissue chopper (McIlwain Tissue Chopper, Mickle Laboratory, Guildford, UK). The fragments were incubated in Dulbecco’s PBS with Ca^2+^and Mg^2+^ (Gibco, Thermo Fisher Scientific, Ghent, Belgium), in the presence of 0.28 Wünsch units/mL Liberase DH (Roche Diagnostics, GmbH, Mannheim, Germany) for 75 minutes in a water bath (37°C) with gentle agitation, and pipetted every 15 minutes. Digestion was terminated by the addition of the same volume of PBS (without Ca^2+^and Mg^2+^) plus 10% of HIFBS (Gibco). The resulting suspension was then centrifuged at 50*g* for 10 minutes at 4°C. The pellet containing follicles was resuspended in PBS + 10% HIFBS and follicles were retrieved with the help of a stereomicroscope (Leica, Van Hopplynus Instruments, Brussels, Belgium) using a 130 μm micropipette (Flexipet, Cook, Limerick, Ireland) at 4°C by two blinded operators.

Immunohistochemistry

*Caspase-3 and Ki67*

Paraffin sections were dewaxed with Histosafe (Yvsolab SA, Beerse, Belgium) and rehydrated in isopropanol (Merck). Endogenous peroxidase activity was blocked by incubation with 0.3% H_2_O_2_ (Merck) diluted in demineralized water (for caspase-3) or 3% H_2_O_2_ diluted in methanol (for Ki67) for 30 minutes at room temperature (RT). After demasking in citrate buffer and Triton X100 (pH 6) for 75 minutes at 98°C, non-specific binding sites were blocked by incubation with normal goat serum for 30 minutes. The sections were then incubated overnight at 4°C with primary antibody, rabbit polyclonal anti-human caspase-3 antibody (G7481, 1:200 dilution, Promega, Madison, USA) or mouse monoclonal anti-human Ki67 antibody (M7240, 1:100 dilution, Dako, Glostrup, Denmark). The slides were subsequently incubated for 60 minutes at RT with goat anti rabbit (K4003, Dako Envision^+^ System HRP) or goat anti-mouse secondary antibody (K4001, Dako Envision^+^ System HRP). Diaminobenzidine (Dako) was used as a chromogen and hematoxylin as a counterstain. Inflamed human tonsil was used as a positive control for caspase-3, and human proliferative endometrium as a positive control for Ki67. Both controls were obtained from our university biolibrary. Negative controls consisted of the dilution solution without any primary antibody.
